# The Integrative Human Microbiome Project: Dynamic Analysis of Microbiome-Host Omics Profiles during Periods of Human Health and Disease

**DOI:** 10.1016/j.chom.2014.08.014

**Published:** 2014-09-10

**Authors:** The Integrative HMP (iHMP) Research Network Consortium

## Abstract

Much has been learned about the diversity and distribution of human-associated microbial communities, but we still know little about the biology of the microbiome, how it interacts with the host, and how the host responds to its resident microbiota. The Integrative Human Microbiome Project (iHMP, http://hmp2.org), the second phase of the NIH Human Microbiome Project, will study these interactions by analyzing microbiome and host activities in longitudinal studies of disease-specific cohorts and by creating integrated data sets of microbiome and host functional properties. These data sets will serve as experimental test beds to evaluate new models, methods, and analyses on the interactions of host and microbiome. Here we describe the three models of microbiome-associated human conditions, on the dynamics of preterm birth, inflammatory bowel disease, and type 2 diabetes, and their underlying hypotheses, as well as the multi-omic data types to be collected, integrated, and distributed through public repositories as a community resource.

## Introduction

The human microbiome is important for human health, behavior, and disease, yet its function and dynamics during healthy and disease states are only partially understood. The first phase of the NIH Human Microbiome Project (HMP, fiscal years 2008–2012, http://www.commonfund.nih.gov/hmp) examined the diversity and composition of the human microbiome to evaluate (1) common patterns of microbial diversity associated with health and (2) whether specific features of the microbiome correlated with specific diseases by analyzing the taxonomic and metagenomic composition of the microbiome in a large healthy cohort and in a set of demonstration projects. These efforts revealed the vast microbial diversity associated with humans and provided new insights into the ecology of the host-microbiome supraorganism (Human Microbiome Project Consortium, 2012a, 2012b).

Results from previous human microbiome studies (Dethlefsen et al., 2006; Hooper and Gordon, 2001; Qin et al., 2010; Savage, 1977) and from the first phase of HMP (Human Microbiome Project Consortium, 2012a, 2012b) suggested that taxonomic composition of the microbiomes between subjects within a cohort can sometimes differ significantly, indicating that taxonomic characterization alone may not be sufficient to reveal relationships between the microbiome and specific health or disease states. Interestingly, metabolic pathway reconstructions of the metagenomic data from the HMP healthy cohort study suggested that the microbiomes of healthy subjects may share similarities in their metabolic pathways; this feature may be a key common property of the microbiome within any given cohort (Human Microbiome Project Consortium, 2012b). Further, in some of the HMP Demonstration Projects (http://commonfund.nih.gov/hmp/fundedresearch), analysis of microbiome biological properties, such as community transcripts, proteins, or metabolites, hinted at losses or gains of key microbiome functions associated with particular diseases.

The second phase of HMP (or iHMP, Integrative Human Microbiome Project Consortium, fiscal years 2013–2016, http://hmp2.org) will further examine the role of the microbiome in human health and disease through a study of three models of microbiome-related human conditions. These three longitudinal studies focus on (1) pregnancy, including those resulting in preterm birth; (2) gut disease onset, using inflammatory bowel disease (IBD) as a model; and (3) respiratory viral infection and onset of type 2 diabetes (T2D). In each case, subjects will be followed for up to 3 years. Multiple properties of the microbiome, such as phylogenetic composition and associated functional omic data (e.g., transcriptome and/or proteome) will be analyzed. Importantly, host functional changes will also be monitored (e.g., transcriptome, proteome, and/or autoantibody profiles) as well as metabolites originating from both the host and the microbiome so that microbiome and host changes can be simultaneously followed to provide a more comprehensive picture of the dynamic changes that occur during these periods. The studies were selected as exemplary models of microbiome-associated human conditions and therefore should be of broad interest to the research community. These resulting data sets will provide opportunities for new hypotheses to be tested and for evaluating the utility of simultaneously assessing the microbiome and the host biological properties for understanding host-microbiome interactions.

## Data Deposition, Access, and Sharing

Primary data (e.g., sequence reads or mass spectrometry profiles) and derived properties (e.g., microbial composition, gene expression and protein levels, and metabolite profiles) will be generated by each project. Table 1 lists the biospecimens and data types to be collected, and the public repositories for the primary data. A glossary for the multi-omic properties is included in Table 2. Primary data will be accessible from public databases such as SRA (genomic sequences, metagenomic sequences, transcript data), GEO (gene expression data), EBI PRIDE (proteomic data), and the Metabolomics Workbench (http://www.metabolomicsworkbench.org/, metabolomic data). Protected data, such as human sequence data and specific clinical metadata, will be deposited in the NCBI dbGaP controlled access database; requests to access these data can be made through dbGaP (https://dbgap.ncbi.nlm.nih.gov). Bacterial strains will be deposited in the HMP reference strain collection at ATCC/BEI. In addition to the public repositories, all nonprotected (i.e., nonhuman) data will also be in structured, study-specific databases hosted by each team. In accordance with HMP policies, all data of sufficiently high quality will be rapidly deposited in public repositories in advance of any iHMP Consortium publication. Analyses will be performed on early data sets from each of the projects; derived data sets used in these analyses will be made available at publication. An iHMP data coordination center (http://hmpdacc.org) will work with the three teams to process common data types in a uniform fashion to optimize data integration across the three studies. A summary of each project as currently proposed and the assays to be executed is presented in the following sections. These clinical studies are under IRB oversight at their respective institutions. All study subjects have been appropriately consented for broad data sharing in open access databases except for protected subject data, which will be deposited in the controlled access database, dbGaP.

## iHMP Study: Integrative Multi-Omic Analysis of the Vaginal and Related Microbiomes in Pregnancy, Including Preterm Birth

The annual United States health care costs for newborns with complications exceeds $26 billion (Behrman and Butler, 2007). Worldwide, preterm birth is the leading cause of morbidity and mortality among neonates. Recent decades have seen improved survival of preterm neonates, but the incidence has not decreased. Very preterm births (<32 weeks), which pose the greatest health risks to the infant, are commonly caused by infection and inflammation in the uterine cavity (Goldenberg et al., 2000). Intrauterine infection can cause premature rupture of membranes, preterm labor, and other complications. Evidence is accumulating that the maternal microbiome undergoes significant changes during pregnancy and plays a key role in fetal and infant morbidity associated with health issues, including but not limited to: gestational diabetes, low birth weight, necrotizing enterocolitis, and colic (Aagaard et al., 2012; de Weerth et al., 2013; Koren et al., 2012; Prince et al., 2014; Romero et al., 2014a, 2014b). Hence, the maternal microbiome likely plays a critical but largely undefined role in the health of the pregnant woman and her fetus.

### Project Description

The goal of the Multi-Omic Microbiome Study: Pregnancy Initiative (http://vmc.vcu.edu/momspi) is to study the dynamic changes in the vaginal and related microbiomes and associated host responses during normal pregnancy, in the development of adverse outcomes of pregnancy, and in early infant microbiome acquisition. It is hypothesized that various components of the microbiome are relevant to maternal, fetal, and neonatal health and that the microbiota contributes to preterm birth and stillbirth. To explore these hypotheses, women will be enrolled early in pregnancy and followed longitudinally at multiple prenatal visits, at admission to and discharge from labor and delivery, and at postpartum visits. Enrollment will be skewed toward populations at high risk for preterm birth. Microbiome compositions will be measured by 16S rRNA gene surveys, whole metagenome shotgun sequencing, and bacteriological cloning and sequencing. Microbial and host gene expression profiles will be examined by whole metatranscriptome sequencing, and cytokine and lipid profiles will be explored. Protein-protein interactomic maps of the microbiomes and the host will also be generated (Table 1). Specifically, this project will:

1. Enroll up to 2,000 pregnant women and their neonates from clinics associated with the VCU Health Center and the Global Alliance to Prevent Prematurity and Stillbirth (GAPPS). Women will be sampled at multiple body sites (vagina, rectum, skin, nares, mouth, blood) longitudinally throughout pregnancy, at labor and delivery, and at follow-up visits. Neonates will be sampled at multiple body sites (rectum, mouth, skin, nares, meconium, stool) at birth and discharge visits. Placenta, cord blood, and chorioamniotic membranes will be collected, and amniotic fluid samples will be collected during cesarean deliveries.
2. Perform multi-omics analyses on maternal and neonatal samples. All samples will be subjected to 16S rRNA gene surveys and all vaginal and serum samples will be subjected to lipidomic analysis. Selected samples will be processed for whole metagenomic and whole metatranscriptomic sequence analysis, host transcriptome profiling, cytokine profiling, bacteriological culture, and protein-protein interaction analyses.
3. Integrate the data from these multi-omic studies into existing and local databases available to vaginal microbiome investigators. The results will reveal associations between the microbiome composition, microbial metabolic potential, gene expression profiles of the host and microbiota, and the host cytokine and metabolome/lipidome expression profiles. Dynamic changes in the microbiomes and in gene expression profiles will be monitored longitudinally during and after pregnancy. Associations with preterm birth, stillbirth, and other adverse outcomes of pregnancy will be identified.

Overall, this research will reveal the impact of the microbiome on the gene expression, cytokine, and metabolome/lipidome profiles of pregnant women and their relationship to preterm birth or other adverse outcomes of pregnancy. The long-term objective of this study is to promote women’s health during pregnancy and prevent adverse outcomes.

### Cohort Description

This study will longitudinally sample about 2,000 women, at least 15 years old, during and after their pregnancies. The neonates of these women will also be sampled. The predicted racial/ethnic composition of our cohort, based on the demographics of visitors to the VCU and GAPPS collection sites, is highly diverse. African Americans, who are at higher risk for preterm birth, represent about one-third of our anticipated cohort. Of the remainder, about 37% will be Hispanic, ~20% Caucasian, ~5% Asian, and ~4% Native American. Because high-risk populations attending high-risk clinics will be sampled, it is anticipated that approximately 15%–20% of the study participants will deliver preterm.

### Sampling

Samples will be collected from pregnant women at 3–5 antepartum visits, at delivery, at discharge, and at subsequent follow-up visits. Neonate samples will be collected at delivery and at discharge. Placenta, cord blood, and amniotic fluid (C-section only) will be collected at delivery. Participants will complete comprehensive electronic surveys that detail health history, background, habits, and diet. These data will be incorporated into a secure database at VCU and a subset will be uploaded to dbGaP.

Vaginal, cervical, rectal, buccal, skin, and nares samples will be collected with soft swabs. Cervical samples will be collected during a speculum exam. Samples from the vagina and rectum and the vaginal pH will be taken at each visit, except discharge, and again at follow-up visits, either by a clinician or by self-sampling. Buccal samples will be collected at each visit, and skin (chest and antecubital fossa) and nares samples at each visit except triage and delivery. Additional skin samples (chest and dominant palm) will be collected at discharge. Blood will be collected from participants early in pregnancy and again at triage. Amniotic fluid will be collected with a sterile syringe, and placenta and membrane samples will be collected with sterile scissors. Cord blood will also be collected.

Buccal, rectal, meconium, stool, skin, and nares samples from term and preterm neonates will be collected with soft swabs at delivery and discharge. Tracheal aspirates will be collected from neonates who are intubated. Over 100,000 samples will be deposited into the RAMS Registry/Repository at VCU (http://www.ramsregistry.vcu.edu) for future research.

### Multi-Omic Assays

**Figure 1:**
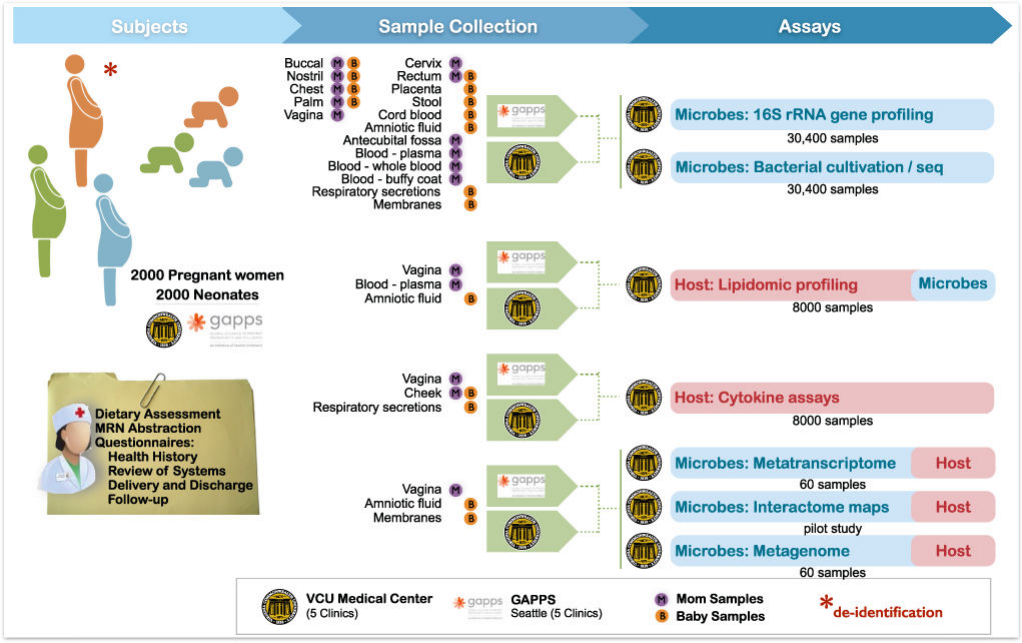
Integrative Multi-Omic Analysis of the Vaginal and Related Microbiomes in Pregnancy: Sample Collection, Assays, and Data Generation Workflow Samples from pregnant women and neonates will be collected at clinics associated with Virginia Commonwealth University and the Global Alliance to Prevent Prematurity and Stillbirth (GAPPS). Health questionnaires will be administered and samples collected from multiple body sites over multiple visits throughout pregnancy, at delivery, at discharge, and at follow-up visits. Neonates will be sampled at delivery, discharge, and follow-up visits. A multi-omic approach will probe properties of the host and microbial communities to generate an integrative, longitudinal, and comprehensive data set of 16S rRNA gene surveys, mass spectrometry-based lipidomic profiles, and cytokine assays. A subset of samples will be subjected to whole metagenome and metatranscriptome sequencin for cultivation and isolation of bacterial strains for genome sequencing, and for generation of interactome maps.

Samples will be subjected to a panel of technologies, including 16S rRNA gene surveys, whole metagenome and metatranscriptome shotgun sequencing, bacterial genome sequencing, cytokine and lipid profiling, and generation of protein-protein interaction networks of the microbiome and the host (Figure 1).

#### 16S rRNA Gene Surveys for Microbiome Profiles: All Sample Types

For 16S rRNA gene survey analysis, DNA will be sequenced in barcoded pools using 2 × 250 bp PE technology on an Illumina MiSeq sequencer in a standard pipeline that generates more than 30,000 reads per sample. Reads will be processed using the RDP Classifier (Wang et al., 2007) and assigned to species using STIRRUPS (Fettweis et al., 2012) or by OTU-based methods to generate microbiome profiles.

#### Whole Metagenome Shotgun Sequencing and Whole Metatranscriptome Sequencing: Vaginal Samples

To clarify the contribution of the metabolic potential and gene expression profiles of the vaginal microbiome to preterm birth, case-matched samples from 60 women who experience preterm birth and 60 women who experience normal term birth will be subjected to in-depth whole metagenome shotgun sequencing and whole metatranscriptome sequencing. Reads will be taxonomically classified and quantified, and metabolic reconstruction and analysis will be performed using the HUMAnN pipeline (Abubucker et al., 2012), ASGARD (Alves and Buck, 2007), or similar tools.

#### Cytokine Profiles: Selected Vaginal and Buccal, Mother and Infant

The immune response will likely undergo dynamic changes during pregnancy, and these may be particularly evident in cases associated with adverse events. To monitor these changes, we will analyze the profiles of a panel of cytokines, including some that were previously linked to preterm birth and/or inflammation (IL-1a, IL-1b, IL-1ra, IL-2, IL-4, IL-6, IL-8, IL-10, IL-12 [p40], IL-12 [p70], IL-17, sCD40L, TNF-α, TNF-β, IFN-γ) in vaginal (mother) and buccal (mother and infant) samples. Representative vaginal and buccal samples from the ~2,000 mothers and infants will be analyzed. Analyses will be performed in a high-throughput format using a multiplex magnetic bead-based human cytokine ELISA assay.

#### Lipidomics: All Vaginal Samples

Bioactive lipids play an important role in the maintenance of pregnancy and the induction of labor. These lipids are likely associated with the local microbiome and pregnancy outcomes. Thus, we plan to analyze levels of more than 150 lipids (e.g., eicosanoids, sphingolipids, and steroid hormones) in vaginal samples collected longitudinally throughout pregnancy using methods previously described (Wijesinghe et al., 2010, 2011). Assays will employ liquid chromatography-tandem mass spectrometry (LC-MS/MS) with a focus on the metabolites that play a role in pregnancy and parturition.

#### Bacterial Genome Sequencing: Selected Samples from All Sites

Many bacteria identified in 16S rRNA surveys of vaginal samples, some of which are associated with women’s urogenital health, have never been cultured or characterized. Several isolates associated with preterm birth (Harwich et al., 2012) have been cultured and characterized, and their pathogenic potential is now being studied. Select isolates cultured from samples with previously unidentified taxa associated with preterm risk will be assayed for pathogenic potential and subjected to whole genome sequence analysis. The assemblies will be deposited into GenBank and the cultures at ATCC/BEI.

#### Interactomics: Protein-Protein Interaction Maps: Selected Vaginal Samples

Microbes colonizing the vagina likely impact vaginal health by secreting proteins that bind to or interact with proteins of cocolonizers or the host. A pilot study will be performed that will apply a novel yeast two-hybrid strategy that invokes high-throughput in silico identification of interacting partners using targeted next-generation sequencing of an amplicon including both bait and prey coding sequences from a special hybrid construct following selection. If the pilot is successful, the technology can be applied to interactions among complex bacterial communities or between bacteria and the host.

## iHMP Study: Characterizing the Gut Microbial Ecosystem for Diagnosis and Therapy in IBD

Over the past decade, IBDs have emerged as one of the most important human conditions linked to the gut microbiota (Abraham and Cho, 2009; Danese and Fiocchi, 2011; Khor et al., 2011; Xavier and Podolsky, 2007). IBD comprises both Crohn’s disease (CD) and ulcerative colitis (UC), which together affect nearly 1.5 million Americans (Loftus, 2004). Studies of environmental (Baumgart and Sandborn, 2012; Molodecky et al., 2011; Ordás et al., 2012; Renz et al., 2011) and microbial (Packey and Sartor, 2009; Sokol et al., 2008) associations with IBD have resulted in neither simple diagnostic markers nor targetable points of intervention. Instead, IBD has been repeatedly linked to the overall ecology of the human gut microbial ecosystem, including community diversity (Frank et al., 2007; Manichanh et al., 2006; Ott et al., 2004), a range of microbial over- and underabundances (Joossens et al., 2011; Scanlan et al., 2006; Sokol et al., 2009; Willing et al., 2009), and the depletion and enrichment of microbial metabolic activities (Erickson et al., 2012; Meyer et al., 2012; Morgan et al., 2012). To date, however, no one aspect of the gut microbiome has yielded data that identify causal biomolecular mechanisms in IBD or provide a translationally actionable target for IBD.

### Project Description

An IBD multi-omic database, the IBDMDB (http://ibdmdb.org), comprising an integrated data resource, will be developed to enable the gut microbial ecosystem as a target for diagnosis, therapy, and mechanistic understanding of IBD. This study will leverage well-phenotyped patient cohorts in both pediatric and adult populations to provide longitudinal profiling of the biological properties of the human gut microbiome in IBD. To further characterize the microbiota’s mechanisms of host interaction, profiles of host genetic and functional properties from a subset of longitudinal time points will also be collected. In addition to data being made rapidly accessible to the community by building on this research group’s current computational infrastructure for multi-omic data processing and distribution, this study will validate and distribute its sample collection and bioinformatic protocols, including platforms for single-cell- and host-cell-focused multi-omic assays.

The biological questions of interest to be addressed by the multi-omic data generated by this project include (1) identifying the molecular mechanisms by which the intestinal microbiome may trigger disease activity in patients with IBD, (2) determining if microbial composition predicts subsequent risk of flares in disease activity, and (3) testing whether successful response to therapy can be predicted from the stool microbiota. These hypotheses are testable by the dense longitudinal multi-omic surveys of the IBD patients and controls in this study design. Specifically, this project will:

1. Create an integrated data repository, the IBDMDB, for standardized community access to multi-omic data, building on existing resources and supporting rapid future algorithm development. This will begin with a pilot data set of existing multi-omic data from two cohorts, including cross-sectional and longitudinal 16S, metagenomic, metabolomic, and proteomic profiles.
2. Implement and validate standardized stool and biopsy sample collection methods among three established IBD patient cohorts.
3. Recruit well-characterized subjects within these three cohorts targeting, respectively, new-onset pediatric subjects, new-onset adult subjects, and established Crohn’s disease and ulcerative colitis, in addition to non-IBD controls.
4. Longitudinally collect stool samples and, for a subset of collection points, rectal and colon biopsies from which microbial 16S surveys, metagenomic and metatranscriptomic sequences, viromes, metabolites, and proteins will be surveyed. A subset of stool and biopsy samples will additionally provide 16S and metagenomic and metatranscriptomic population analyses through single-cell techniques (Table 1).
5. Acquire host information, including rich clinical and phenotypic metadata for all collection points and, for a subset, host genetics, gene expression data, DNA methylation profiles, serology, and primary intestinal epithelial cells for profiling response to microbial products.

### Cohort Description

The study will collect stool samples, biopsies, and blood samples from three clinical cohorts. The primary sample will be serial stools every 2 weeks for 1 year, which will be analyzed using 16S rRNA gene surveys, whole metagenome and metatranscriptome shotgun sequencing, metabolomics, whole virome shotgun sequencing, proteomics, and a fecal calprotectin assay. Intestinal biopsies will also be collected at a standardized rectal location for all subjects, plus at the site of active inflammation for IBD patients, at time zero and as clinically indicated over the following year (approximately one additional time point per subject). 16S surveys will be carried out on these biopsies, in addition to host whole transcriptome sequencing and host cell profiling by culture. A subset of biopsies and stools will be additionally assessed by single-cell 16S and whole metagenome and metatranscriptome shotgun sequencing. Finally, blood samples will be drawn at recruitment and 6 month intervals for host genetics, serology, and epigenetics by reduced representation bisulfite sequencing (RRBS).

This study includes three IBD cohorts providing these samples and metadata, coupled with two additional cohorts from which early “proof-of-principle” data are already available. The samples described above will be collected from (1) PRISM (the Prospective Registry in IBD Study at MGH), recruiting from Massachusetts General Hospital (Ananthakrishnan et al., 2014; Morgan et al., 2012); (2) the MLI (Mucosal Luminal Interface) cohort recruiting from Cedars-Sinai Medical Center (McHardy et al., 2013; Tong et al., 2013); and (3) a pediatric IBD cohort recruiting from Emory University and Cincinnati Children’s Hospital (Gevers et al., 2014; Walters et al., 2014). Each cohort will provide 30 subjects (12 CD, 12 UC, and 6 non-IBD controls), with MGH focusing on new-onset adult patients, CSMC on adult patients with established disease, and Emory/Cincinnati on new-onset pediatric patients. Together, these cohorts have previously enrolled a total of over 3,600 subjects and collected more than 1,500 samples. A smaller, existing multi-omic data set is included in this study as a proof-of-principle, derived from two Swedish cohorts, the Swedish Twin and Swedish Longitudinal studies of IBD (Dicksved et al., 2008; Erickson et al., 2012; Willing et al., 2010). These include up to 40 twin pairs and 119 individuals providing up to ten time points, respectively, and represent one of the only existing multi-omic data sets in IBD around which the computational infrastructure will be organized and prototyped.

### Sampling

This study has already established a convenient method for self-collected fecal samples compatible with all of the multi-omic assays, enabling longitudinal profiling in smaller collection tubes tolerant of over 24 hr at ambient temperature (Franzosa et al., 2014). Two tubes are provided per subject, one dry (for proteomics and calprotectin) and the other containing an ethanol fixative (for all other assays). Validation studies indicate that there is close agreement between freshly frozen fecal sample assays and samples in preservative. Each fecal collection tube cap includes a spoon for easier collection and can be shipped under ambient conditions, and clear instructions are provided to the subjects (publicly available at http://ibdmdb.org/protocols). Each sample is shipped by subjects in a self-addressed, prepaid FedEx box for same-day delivery, with aliquots distributed in subsequent batches for off-site assays. Samples are accompanied by short metadata questionnaires, and clinical data at first visit includes demographics, disease characterization (including medication history), short- and long-term food frequency questionnaires, and an environmental factors questionnaire.

### Multi-Omic Assays

#### Microbial Community Composition, Functional Potential and Activity: All Samples

**Figure 2:**
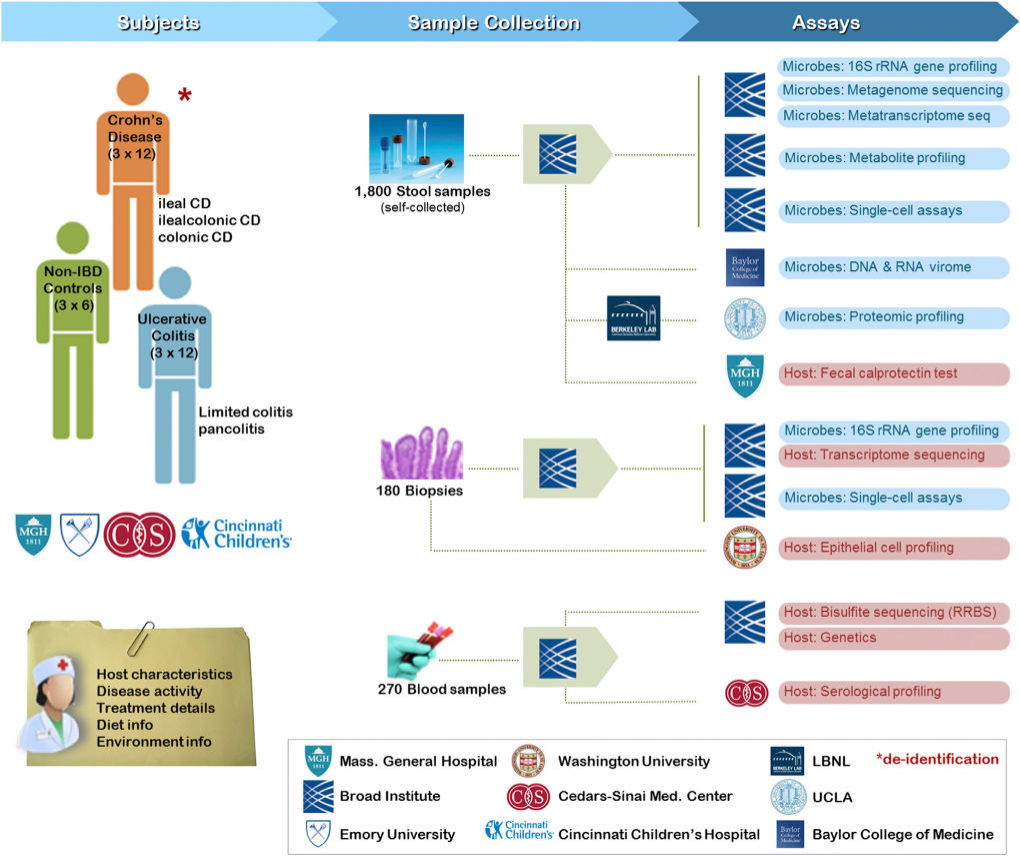
Characterizing the Gut Microbial Ecosystem for Diagnosis and Therapy in Inflammatory Bowel Disease: Sample Collection, Assay, and Data Generation Workflow Samples from Crohn’s disease patients, ulcerative colitis patients, and non-IBD controls are collected at Massachusetts General Hospital (adult new onset), Emory University (pediatric new onset), Cincinnati Children’s Hospital (pediatric new onset), and Cedars-Sinai Medical Center (adult established). From each patient, three different types of samples are collected: longitudinal stool samples, periodic biopsies, and regularly scheduled blood samples. Biopsies are collected as clinically indicated, blood during clinical visits, and stool samples are self-collected by participants at home and shipped directly to a centralized handling and aliquotting pipeline. Multi-omic data generation (primarily, but not entirely, nucleotide sequence- and mass spectroscopy-based) provides microbial, host, and mixed profiles including 16S rRNA gene surveys, whole metagenome and metatranscriptome shotgun sequences, metabolite and protein profiles, single-cell assays, whole virome shotgun sequences, and serological profiles. Each sample is further accompanied by clinical (bloods/biopsies) or self-reported environmental and dietary (stools) metadata.

The first tier of multi-omic data in IBD (Figure 2) will include 16S rRNA surveys, whole metagenomic shotgun sequencing, and whole metatranscriptome shotgun sequencing from stool samples (all assays) and biopsies (16S only). Improving our understanding of complex human-associated microbial communities will require at a minimum the aspects of microbial community structure, its functional potential, and realized gene expression activity. This dictates the inclusion of 16S rRNA gene-based, whole metagenome DNA shotgun-based, and community mRNA-based sequencing (Gevers et al., 2012). This study will also survey the nonbacterial members of the gut microbiota, including archaeal, viral, and eukaryotic components. This study includes a viral enrichment and sequencing protocol for each stool sample.

#### Metabolomic and Protein Profiles: Stool Samples

This study will measure metabolomic and protein profiles of stool samples from participants in the project cohorts using a combination of mass spectrometry (MS) methods. Four LC-MS/MS approaches will measure complementary metabolite classes using sensitive, high-resolution mass spectrometers enabling nontargeted measurements of known metabolites (several hundred in total) and unidentified metabolites in the same run. For each stool sample, metabolites will be identified using reference data generated from an in-house Broad Institute library of > 500 compounds, and novel metabolites will be annotated using a database of > 8,000 metabolites and confirmed using references when possible.

Protein profiles will be measured by two-dimensional nano liquid chromatography (2D-LC) and tandem mass spectrometry (MS/MS) (Cantarel et al., 2011; Presley et al., 2012), with proteins identified by searching custom databases consisting of the human genome, matched metagenomes from the same samples, and a defined set of reference genomes, including many that are in the HMP reference genome database. Based on preliminary results, it is anticipated that > 3,000 proteins will be identified per sample and that approximately half of the proteins will be human proteins based on a pretested method for preparation of the protein extracts. The combination of human and microbial protein data will provide a window into the types of host-microbe interactions that occur in the gut and how these functions are correlated to IBD.

#### Host Biomolecular Activities in the Gut: Colon Biopsy Samples and Cultured Host Cells

To begin linking gut microbial community biology to that of the host, this study will assess changes in host biomolecular activities in the gut as part of multi-omic profiling. Specifically, host gene expression will be assayed by RNA-seq from colon biopsies (from a standardized rectal location in all subjects and from the active site of IBD in patients) and the DNA methylation state of the host genome in circulating blood by RRBS. This will allow the discovery of links between epigenetic content and disease states relating to microbial communities. Finally, to detail pathway-level crosstalk between gut microbes and the intestinal epithelium, host cells isolated from primary biopsy samples will be cultured and perturbed in vitro (Gallo and Hooper, 2012; Maynard et al., 2012; Stappenbeck et al., 1998) to determine the relative contribution of metabolites and host epithelial cells in this crosstalk.

## iHMP Study: Microbiome and Host Changes during Respiratory and Other Stress Conditions in Individuals at Risk for T2D

Type 2 diabetes mellitus (T2D) is a significant health problem facing our nation. Close to 20 million individuals in the United States have T2D, costing hundreds of billions of dollars annually, and the incidence is projected to double by 2050 (American Diabetes Association, 2008). Differences in the gut microbiome have been noted between diabetics and healthy individuals (Qin et al., 2012), and direct alteration of the microbiome in mice has been shown to lower glucose levels (Turnbaugh et al., 2006). The hypotheses to be tested in this study include the following: (1) environmental stress causes dynamic changes in specific biological pathways in the human body, and these changes lead to alteration of the human microbiome and the metabolome, including glucose; (2) some of the changes may affect the epigenome, leading to molecular and biological alterations that extend well beyond the time of the stress period; and (3) different physiological stresses, such as respiratory viral infections and diet changes, may have common effects in both the host and microbiome.

### Project Description

To better understand the biological changes that occur during the onset and progression of T2D, this study (http://med.stanford.edu/ipop/) will perform a detailed analysis of the biological processes that occur in the human microbiome and host by longitudinal profiling of patients at risk for T2D during healthy and stress periods. Multiple sample types will be collected from the study participants every 2–3 months during their healthy periods, with more frequent sampling during periods of respiratory illness and other environmental stress. Multiple microbiome biological properties will be analyzed. Changes in host functional profiles will also be revealed.

This study is designed to develop a detailed understanding of the physiological changes that occur in the microbiome and host during viral infection and during changes in glucose levels and insulin resistance. This longitudinal study is expected to reveal changes in the microbiome and host at an unprecedented level and frequency and to identify the molecules and pathways that change during viral infections and diabetes onset and progression. Specifically, this project will:

1. Establish a cohort of approximately 60 consented individuals at risk for T2D. Dense sampling will occur during respiratory infection and other stress states, with less frequent sampling every 2–3 months during healthy periods,
2. Perform multi-omic analysis of longitudinal microbiome and host samples (Table 1). Microbiome genomic, transcriptome, and proteome content will be determined in fecal and nasal samples and in peripheral blood monocyte cell (PBMC) samples for viral and endogenous microbiome populations. Host genomic, transcriptomic, and proteomic data will be concurrently analyzed in blood components (PBMCs for genome and transcriptome; plasma for proteome). Combined microbiome and host metabolites will be analyzed in serum and urine. Antivirome antibodies will also be analyzed.
3. Analyze and integrate omic information from the microbiome and host. The different omics information (genome, transcriptome, and proteome) from nasal and fecal samples will be analyzed individually and in a combined fashion to determine the dynamic pathways that change during viral infection. In addition this study will correlate microbiome omic temporal profiles with those of the host to obtain an unprecedented view of the global microbiome-host changes that occur during viral infections.

### Cohort Description

This study will execute a 3 year study in 60 males and females at high risk for T2D. Those individuals with either impaired fasting glucose (fasting plasma glucose > 100 mg/dl) or impaired glucose tolerance (2 hr glucose > 140 mg/dl in oral glucose tolerance test) will be enrolled. In order to further increase the number of study participants with chances of conversion to diabetes, but to maintain some degree of homogeneity in study subjects, the study will include only those volunteers with body mass index 25–40 kg/m^2^ and age 35–65 years. Given estimates of 11% conversion to frank diabetes per year (Ratner and Diabetes Prevention Program Research, 2006), the study anticipates up to ten cases of diabetes to occur over the 3 year study period. All participants will receive genetic counseling prior to enrollment.

### Sample and Data Collection

Qualified and consented participants will proceed to baseline testing, which includes a blood draw for molecular omics analysis, as well as measurement of fasting lipid panel markers HbA1c and hsCRP. Subjects will return every 3 months for blood draws and microbiome samples and will complete online surveys that are automatically sent to them to document changes in medications, activity, body weight, blood pressure, diet, menstrual cycle, stresses, etc. Subjects will undergo frequent sampling during periods of environmental or medical stress. In this study, stresses are defined as medical illness, physical injury/pain, major or minor operation, major life changes (birth, death, divorce, marriage, and change of home or job). The study will reinforce different collection schemes depending on types of stress events; for example, in cases of viral infection, the study will collect samples on days 1, 2, 4, 7, 21, and 35 after the onset of infection symptoms.

For each visit, either quarterly healthy visit or during stress periods, the study will collect peripheral blood to prepare PBMCs, plasma and serum, as well as urine and microbiome samples (nasal, fecal, skin, and tongue) using standard HMP protocols.

### Multi-Omic Assays

**Figure 3:**
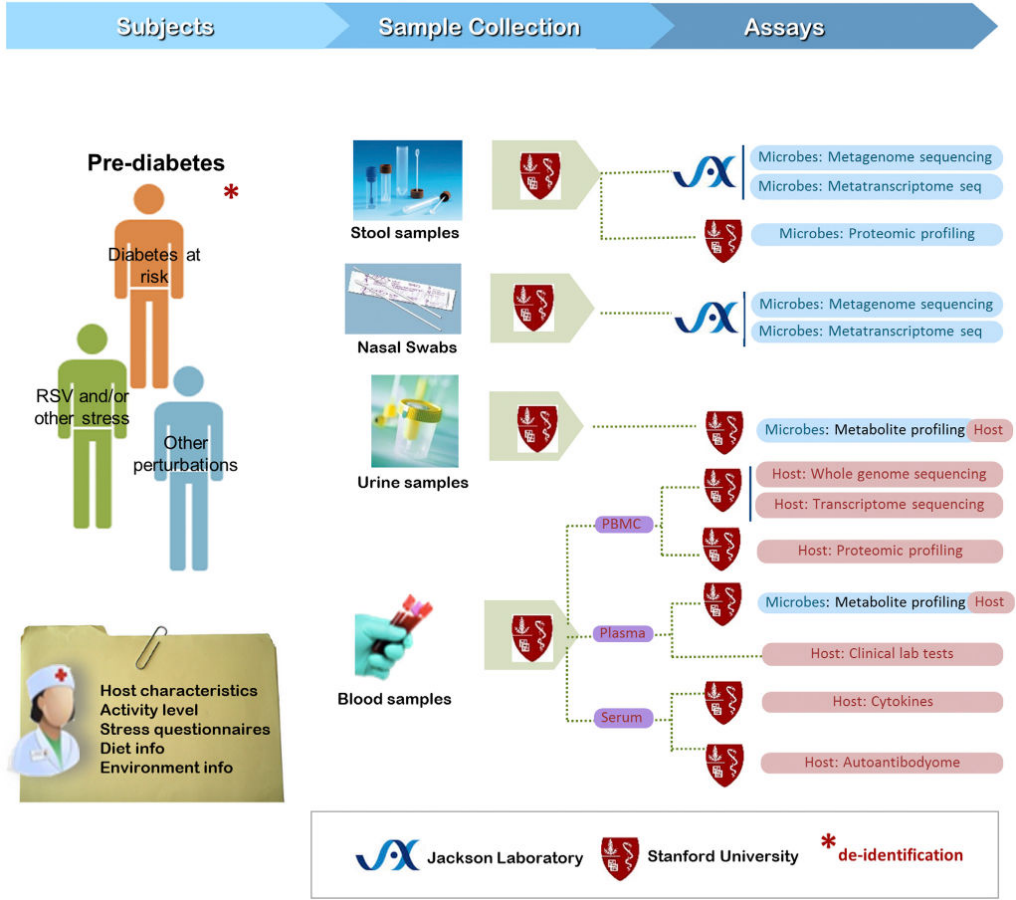
Microbiome and Host Changes during Respiratory and Other Stress Conditions in Individuals at Risk for Type 2 Diabetes: Sample Collection, Assay, and Data Generation Workflow All samples are collected at the Stanford Clinical & Translational Research Unit. From each patient in every visit, blood sample and microbiome sample (including nasal swabs and stool and urine samples) are collected. Multi-omic data generation (primarily, but not entirely, nucleotide sequence- and mass spectroscopy-based) will provide profiles of microbial phylogenetic composition, metagenomes, metatranscriptomes, and metaproteomes; host protein profiles, cytokines, and autoantibodies; and global metabolome profiles. Each sample is further accompanied by clinical (blood) or self-reported stress level, environmental, and dietary (stool and urine) metadata.

To better understand the relationship between healthy and stressed states in this cohort, the study will be analyzing a suite of omic properties from both the host and microbiome. These include whole metagenome shotgun and metatranscriptome sequencing, host whole genome/transcriptome sequencing, cytokine and autoantibody profiles, metabolomics profiles, and standard clinical tests (Figure 3).

#### Whole Metagenome Shotgun and Metatranscriptome Sequencing: Stool and Nasal Samples

To follow the diversity of the microbiome in prediabetics and how it changes during stress periods, both 16S and whole metagenome shotgun sequencing of nucleic acids isolated from nasal and stool samples will be performed. Previously established HMP methods for alignment of whole metagenomic shotgun sequences to reference genomes will be used (Human Microbiome Project Consortium, 2012a, 2012b; Martin et al., 2012) for computation of taxonomic representation and breadth and depth of coverage, for variant calling in metagenomic data (Schloissnig et al., 2013), for pathway analysis (Abubucker et al., 2012), and for virome analysis (Wylie et al., 2012a, 2012b).

To follow microbiome activity, microbial gene expression will be analyzed. Bacterial RNA will be isolated and enriched for mRNA using procedures that remove > 95% of 16S and 23S rRNAs (Maurice et al., 2013; Turnbaugh et al., 2009). The resulting RNA is converted into cDNA using random priming and sequenced using the Illumina platform. Analysis of gene expression for each gene will follow previously published computational pipelines and algorithms (Maurice et al., 2013; Turnbaugh et al., 2009).

#### Host Whole Genome/Transcriptome Sequencing: PBMCs

To better understand how each subject responds to dynamic changes as well as to facilitate mapping for downstream transcriptome and proteome analyses, participants will have their genome sequenced (from PBMCs) using Illumina technology. Single nucleotide variants, short insertions and deletions (<100 bp Indels), and structural variants will be scored using the HugeSeq pipeline (Lam et al., 2012). For each PBMC sample, the transcriptome will be analyzed using RNA-seq, involving the random primed synthesis of cDNA from RNA depleted of rRNA. The reads will be mapped, and gene expression, isoform expression, allele expression, and RNA editing will be mapped using a previously published pipeline (Chen et al., 2012).

#### Microbiome and Host Protein Expression Profiles Using LC-MS/MS: Fecal and PBMC Samples

Most studies analyze RNA expression; however, protein levels often do not correlate well with RNA expression levels (Maier et al., 2009). Therefore, microbial and host protein expression will also be analyzed from fecal samples and host PBMC cells, respectively. Proteins will be extracted, trypsin digested, and analyzed by 2D-LC and MS/MS (Chen et al., 2012). MS/MS spectra will be searched using the Sequest algorithm against human and microbiome protein databases and a specialized database of each individual based on the microbiome metagenome and host genome sequence. Protein false discovery rates of 1% and only proteins with at least two peptides will be used. Relative protein levels using TMT isobaric tags and a reference sample prepared from multiple individuals or time points will be quantified (Chen et al., 2012). The dynamic changes in protein levels will be scored during healthy and stress periods.

#### Cytokines and Autoantibody Profiles: Serum

Since inflammation and immune responses have been implicated in T2D (Esser et al., 2014; Shu et al., 2012), and viral infections dramatically affected the immune response, the levels of 50 diverse cytokines (e.g., inflammatory proteins such as TNF-α, IFN-α, CRP, and insulin peptides) in host serum will be followed using throughput ELISA assays (Chen et al., 2012). Autoantibodies and antiviral antibodies will be profiled from selected sera using the Invitrogen ProtoArray Protein Microarray v5.0, which contains 9,483 unique human proteins (Hudson et al., 2007), and other comprehensive human and virome arrays.

#### Metabolomes: Urine and Plasma

Since diabetes is a metabolic disease (Akash et al., 2013), it is of particular importance to profile as many metabolites as possible (Friedrich, 2012), many of which are likely synthesized or influenced by the microbiome. To gain insight into the breadth of metabolic changes that occur, untargeted metabolomics profiling of urine and plasma samples will be performed using an optimized LC-MS procedure (liquid chromatography coupled to mass spectrometry). Two complementary separation methods will be combined and run in ESI (electrospray ionization) positive and negative modes to maximize the metabolome coverage from polar to nonpolar metabolites: HILIC (hydrophilic interaction liquid chromatography) and RPLC (reverse-phase liquid chromatography). The MS data will be acquired using high-sensitivity, high-resolution mass spectrometers. The optimized analytical procedure will monitor 18,000 and 14,000 metabolic features, characterized by a unique retention time and exact mass, for each urine and plasma sample, respectively. Metabolites will then be annotated using public databases of > 40,000 metabolites, and their identity will be confirmed with authentic standards when possible.

#### Standard Clinical Tests: Plasma and Serum

Standard clinical tests will be performed to follow typical plasma and serum markers. We will quantify lipids, lipoproteins, hsCRP, hemaglobinA1c, insulin, and glucose. The degree of hyperglycemia and insulinemia and insulin resistance of subjects will be measured. When patients are ill, standard nasal swabs will be used to identify the pathogen, which will complement the microbiome analyses. The clinical data may be redundant with the omics profiles presented above but will both serve as useful controls and ensure that no key metabolite is missed. In order to document behavior changes of patients, emotional and psychological stress will be quantified using the Perceived Stress Scale instrument, which measures an individual’s perception of stress (Cohen et al., 1983). Physical activity will be scored using the International Physical Activity Questionnaire (Hallal and Victora, 2004), and food intake will be recorded for the last 24 hr before each visit.

## Conclusion

These three examples of microbiome-associated human conditions will provide innovative, freely available, multi-omic data of properties analyzed in both the microbiome and host with which to explore the behavior of the human microbiome over the course of complex conditions using preterm birth, onset of IBD, and onset of T2D as dynamic model systems. In addition, they will help lay the foundation for shared protocols and analysis methods for highly detailed studies of the microbiome’s basic biology and translational potential. Data produced by the iHMP Consortium will be routinely released, and ongoing feedback is encouraged on how best to leverage these resources. For those interested in participating in the iHMP Consortium or learning more about the iHMP data sets or tools, please either contact the corresponding author or send an inquiry via the iHMP portal (http://hmp2.org).

## Supplementary Material

1

## Figures and Tables

**Table 1:** Summary of Biospecimens, Primary Data, and Derived Properties to Be Collected for Each iHMP Cohort Study with Repositories for Primary Data

Source of Property	Property Derived from Primary Data	Primary Data from Biospecimen	Biospecimen from Preterm Birth Cohort	Biospecimen from IBD Cohort	Biospecimen from Prediabetic Cohort	Repository for Primary Data
Microbiome	microbial community composition	16S rRNA gene survey	cervical^a^, vaginal^a^, rectal, buccal, fetal membranes, placenta, amniotic fluid from women; buccal, rectal, stool, meconium, respiratory secretions from neonate	stool	anterior nares, stool	SRA
	microbial community composition	whole metagenome shotgun sequences	vaginal^a^	stool	anterior nares, stool	SRA
	predictions of microbial genes, metabolic pathways	whole metagenome shotgun sequences	vaginal^a^	stool	anterior nares, stool	SRA
	RNA transcript profiles	whole metatranscriptome shotgun sequences	vaginal^a^	stool	anterior nares, stool	SRA
	microbiome metaproteome profiles	LC-MS/MS peptide profiles	–	stool	stool	EBI PRIDE and/or Peptide Atlas
	viral community composition	whole virome shotgun sequences	–	stool	anterior nares, stool	SRA
	bacterial cultures	bacterial isolates	cervical^a^, vaginal^a^, rectal, buccal, stool from mothers or neonates	–	–	ATCC/BEI
	bacterial whole genome sequences	bacterial isolates	cervical^a^, vaginal^a^, rectal, buccal, stool from mothers or neonates	–	–	SRA
	bacterial whole genome sequences	bacterial single-cell sequences	–	stool	–	SRA
	single-cell bacterial RNA transcript profiles	single-cell bacterial transcript sequences	–	stool	–	SRA
Host	subject exome/whole genome	subject genome sequences	blood (future^b^) from mothers and neonates	blood	blood	dbGaP/SRA
	RNA transcript profiles	whole transcriptome sequences	vaginal^a^	colon biopsy	PBMCs	dbGaP/SRA and GEO
	subject protein profiles	LC-MS/MS peptide profiles	–	stool	PBMCs, serum (future)	EBI PRIDE and/or Peptide Atlas
	systemic inflammation levels	cytokine profiles	vaginal^a^, buccal from mothers or neonates	blood	plasma	Study DB
	intestinal epithelial cell cultures	intestinal epithelial cell isolates	–	colon biopsy	–	–
	subject DNA methylation profiles	reduced representation bisulfite sequencing (RRBS) profiles	–	blood	PBMC (future)	SRA
	intestinal inflammation levels	fecal calprotectin protein concentrations	–	stool	–	Study DB
	serum antibody composition and levels	serology	–	blood	–	Study DB
	subject contaminating genome sequences	human sequence from unfiltered total microbial community sequences	vaginal^a^	stool	stool	dbGaP/SRA
	subject profiles for cohort	subject phenotypes, clinical metadata, medical panels	collected on each subject in the study	collected on each subject in the study	collected on each subject in the study	dbGaP
Global (host and microbiome)	protein-protein interaction network between host and microbiome	yeast two-hybrid binary protein complexes	vaginal^a^	–	–	EBI IntAct
	pathway-level crosstalk between host and gut microbiome	intestinal epithelial cell profiling response to bacterial metabolites	–	colon biopsy	–	dbGaP/SRA and GEO
	global metabolite profiles	untargeted and targeted LC-MS metabolomic profiles	–	stool	urine, plasma	Metabolomics Workbench
	global lipid profiles	untargeted and targeted LC-MS metabolomic profiles	vaginal^a^	stool	urine, serum	Metabolomics Workbench

a From mothers only

b Samples collected for possible future analysis

**Table 2:** Glossary of Terms Used to Describe Primary Data in the Paper, in the Figures, and in Table 1.

Primary Data Type	Definition
16S rRNA gene survey	Sequence-based analysis of 16S ribosomal RNA gene in total DNA extracts; the data are used to develop microbial community compositional profiles.
Whole metagenome shotgun sequences	Sequence-based analysis of all genes in total DNA extracts; the data are used to develop microbial community compositional, functional, and genomic profiles.
Whole metatranscriptome shotgun sequences	Sequence-based analysis of RNA transcripts in a microbial community by converting RNA in total RNA extracts to complementary DNA and sequencing the cDNA.
Whole virome shotgun sequences	Sequence-based analysis of all virus genes and genomes used to develop viral community profiles. These sequence data are derived from two approaches, either by first isolating viral-sized particles from a sample and then sequencing this fraction, or by sequencing the DNA (or cDNA from RNA) extract and computationally determining DNA and RNA viral sequences in the metagenome.
Bacterial isolates	Isolation and cultivation of a specific bacterium in a mixed community through the use of selective media or enrichment techniques to preferentially grow one bacterium of interest.
Single-cell genome sequences	Physical separation or physical enrichment of a single microbial cell from a mixed population of cells. Sequence-based analysis of the single cell’s genome is conducted using a DNA random priming method to first increase the total DNA concentration in the cell and subsequently sequence this amplified DNA.
Single-cell RNA sequences	Sequence-based analysis of RNA transcripts in a cell by converting RNA in total RNA extracts to complementary DNA and sequencing the cDNA.
LC-MS/MS peptide profiles	Analysis of individual peptides in a mixture of proteins by a combination of liquid chromatography and mass spectroscopy after enzymatic digestion of the protein mixture. To derive the microbiome metaproteome, data from the companion metagenome is used along with KEGG and other protein databases to verify the microbial proteins in the total protein mixture. To derive the host proteins, a similar approach is used based on search against the human genome.
Human subject whole genome sequences	Analysis of the sequence of the host genome using high-throughput DNA sequencing.
Whole exome sequences	Analysis of the protein coding regions of the host genome, which is generally done through sequence analysis.
Whole transcriptome sequences	Sequence-based analysis of RNA transcripts in host tissue using polyA tail separation of eukaryotic transcripts from a mixture of transcripts, converting the RNA to complementary DNA and sequencing the cDNA.
Cytokines	Circulating immune system glycoproteins in blood, plasma, or other bodily fluids are measured through an ELISA assay; data are used as a marker for systemic inflammation.
DNA methylation profiles	Measurement of methylated regions of the host genome. This method uses a combination of restriction enzymes and a reduced representation bisulfite sequencing (RRBS) method to enrich for regions of the genome with cytosine-guanine pairs; these regions are then screened for methylated bases.
Fecal calprotectin proteins	Fecal calprotectin is a protein found in stool, the concentration of which is measured through a standardized immunoassay method and is used to evaluate levels of intestinal inflammation.
Serology	Measurement of antibodies for specific pathogens or protein markers conducted with serum through an ELISA assay.
Human subject contaminating genome sequences	Sequence from total nucleotide extracts from most microbially focused sample types yields a combination of host and microbial sequence data. A computational filtering step, using human genome reference sequence, will separate microbiome from human sequences.
Interactomes	Analysis of protein-protein interactions between members of the microbiota or between the microbiota and the host.
Intestinal epithelial cell profiles	Functional or sequence-based readouts of phenotypes for cell lines derived from primary epithelial cells from individual hosts.
Metabolomes	Measure of metabolomic profiles using untargeted and targeted LC-MS methods.
Lipidomes	Measure of lipid profiles using 2D UPLC-ESI-MS/MS (ultra performance liquid chromatography combined with electrospray ionization tandem mass spectroscopy) with an initial focus on AA (arachidonic acid) and eicosanoids.

## References

[R1] Aagaard K, Riehle K, Ma J, Segata N, Mistretta TA, Coarfa C, Raza S, Rosenbaum S, Van den Veyver I, Milosavljevic A (2012). A metagenomic approach to characterization of the vaginal microbiome signature in pregnancy. PLoS ONE.

[R2] Abraham C, Cho JH (2009). Inflammatory bowel disease. N Engl J Med.

[R3] Abubucker S, Segata N, Goll J, Schubert AM, Izard J, Cantarel BL, Rodriguez-Mueller B, Zucker J, Thiagarajan M, Henrissat B (2012). Metabolic reconstruction for metagenomic data and its application to the human microbiome. PLoS Comput Biol.

[R4] Akash MS, Rehman K, Chen S (2013). An overview of valuable scientific models for diabetes mellitus. Curr Diabetes Rev.

[R5] Alves JM, Buck GA (2007). Automated system for gene annotation and metabolic pathway reconstruction using general sequence databases. Chem Biodivers.

[R6] American Diabetes Association (2008). Economic costs of diabetes in the U.S. In 2007. Diabetes Care.

[R7] Ananthakrishnan AN, Huang H, Nguyen DD, Sauk J, Yajnik V, Xavier RJ (2014). Differential effect of genetic burden on disease phenotypes in Crohn’s disease and ulcerative colitis: analysis of a North American cohort. Am J Gastroenterol.

[R8] Baumgart DC, Sandborn WJ (2012). Crohn’s disease. Lancet.

[R9] Behrman RE, Butler AS (2007). Preterm Birth: Causes.

[R10] Cantarel BL, Erickson AR, VerBerkmoes NC, Erickson BK, Carey PA, Pan C, Shah M, Mongodin EF, Jansson JK, Fraser-Liggett CM, Hettich RL (2011). Strategies for metagenomic-guided whole-community proteomics of complex microbial environments. PLoS ONE.

[R11] Chen R, Mias GI, Li-Pook-Than J, Jiang L, Lam HY, Chen R, Miriami E, Karczewski KJ, Hariharan M, Dewey FE (2012). Personal omics profiling reveals dynamic molecular and medical phenotypes. Cell.

[R12] Cohen S, Kamarck T, Mermelstein R (1983). A global measure of perceived stress. J Health Soc Behav.

[R13] Danese S, Fiocchi C (2011). Ulcerative colitis. N Engl J Med.

[R14] de Weerth C, Fuentes S, de Vos WM (2013). Crying in infants: on the possible role of intestinal microbiota in the development of colic. Gut Microbes.

[R15] Dethlefsen L, Eckburg PB, Bik EM, Relman DA (2006). Assembly of the human intestinal microbiota. Trends Ecol Evol.

[R16] Dicksved J, Halfvarson J, Rosenquist M, Järnerot G, Tysk C, Apajalahti J, Engstrand L, Jansson JK (2008). Molecular analysis of the gut microbiota of identical twins with Crohn’s disease. ISME J.

[R17] Erickson AR, Cantarel BL, Lamendella R, Darzi Y, Mongodin EF, Pan C, Shah M, Halfvarson J, Tysk C, Henrissat B (2012). Integrated metagenomics/metaproteomics reveals human host-microbiota signatures of Crohn’s disease. PLoS ONE.

[R18] Esser N, Legrand-Poels S, Piette J, Scheen AJ, Paquot N (2014). Inflammation as a link between obesity, metabolic syndrome and type 2 diabetes. Diabetes Res Clin Pract.

[R19] Fettweis JM, Serrano MG, Sheth NU, Mayer CM, Glascock AL, Brooks JP, Jefferson KK, Buck GA, Vaginal Microbiome Consortium (additional members) (2012). Species-level classification of the vaginal microbiome. BMC Genomics.

[R20] Frank DN, St Amand AL, Feldman RA, Boedeker EC, Harpaz N, Pace NR (2007). Molecular-phylogenetic characterization of microbial community imbalances in human inflammatory bowel diseases. Proc Natl Acad Sci USA.

[R21] Franzosa EA, Morgan XC, Segata N, Waldron L, Reyes J, Earl AM, Giannoukos G, Boylan MR, Ciulla D, Gevers D (2014). Relating the metatranscriptome and metagenome of the human gut. Proc Natl Acad Sci USA.

[R22] Friedrich N (2012). Metabolomics in diabetes research. J Endocrinol.

[R23] Gallo RL, Hooper LV (2012). Epithelial antimicrobial defence of the skin and intestine. Nat Rev Immunol.

[R24] Gevers D, Knight R, Petrosino JF, Huang K, McGuire AL, Birren BW, Nelson KE, White O, Methé BA, Huttenhower C (2012). The Human Microbiome Project: a community resource for the healthy human microbiome. PLoS Biol.

[R25] Gevers D, Kugathasan S, Denson LA, Vázquez-Baeza Y, Van Treuren W, Ren B, Schwager E, Knights D, Song SJ, Yassour M (2014). The treatment-naive microbiome in new-onset Crohn’s disease. Cell Host Microbe.

[R26] Goldenberg RL, Hauth JC, Andrews WW (2000). Intrauterine infection and preterm delivery. N Engl J Med.

[R27] Hallal PC, Victora CG (2004). Reliability and validity of the International Physical Activity Questionnaire (IPAQ). Med Sci Sports Exerc.

[R28] Harwich MD, Serrano MG, Fettweis JM, Alves JM, Reimers MA, Buck GA, Jefferson KK, Vaginal Microbiome Consortium (additional members) (2012). Genomic sequence analysis and characterization of Sneathia amnii sp.nov. BMC Genomics.

[R29] Hooper LV, Gordon JI (2001). Commensal host-bacterial relationships in the gut. Science.

[R30] Hudson ME, Pozdnyakova I, Haines K, Mor G, Snyder M (2007). Identification of differentially expressed proteins in ovarian cancer using high-density protein microarrays. Proc Natl Acad Sci USA.

[R31] Human Microbiome Project Consortium (2012a). A framework for human microbiome research. Nature.

[R32] Human Microbiome Project Consortium (2012b). Structure, function and diversity of the healthy human microbiome. Nature.

[R33] Joossens M, Huys G, Cnockaert M, De Preter V, Verbeke K, Rutgeerts P, Vandamme P, Vermeire S (2011). Dysbiosis of the faecal microbiota in patients with Crohn’s disease and their unaffected relatives. Gut.

[R34] Khor B, Gardet A, Xavier RJ (2011). Genetics and pathogenesis of inflammatory bowel disease. Nature.

[R35] Koren O, Goodrich JK, Cullender TC, Spor A, Laitinen K, Bäckhed HK, Gonzalez A, Werner JJ, Angenent LT, Knight R (2012). Host remodeling of the gut microbiome and metabolic changes during pregnancy. Cell.

[R36] Lam HY, Pan C, Clark MJ, Lacroute P, Chen R, Haraksingh R, O’Huallachain M, Gerstein MB, Kidd JM, Bustamante CD, Snyder M (2012). Detecting and annotating genetic variations using the HugeSeq pipeline. Nat Biotechnol.

[R37] Loftus EV (2004). Clinical epidemiology of inflammatory bowel disease: Incidence, prevalence, and environmental influences. Gastroenterology.

[R38] Maier T, Güell M, Serrano L (2009). Correlation of mRNA and protein in complex biological samples. FEBS Lett.

[R39] Manichanh C, Rigottier-Gois L, Bonnaud E, Gloux K, Pelletier E, Frangeul L, Nalin R, Jarrin C, Chardon P, Marteau P (2006). Reduced diversity of faecal microbiota in Crohn’s disease revealed by a metagenomic approach. Gut.

[R40] Martin J, Sykes S, Young S, Kota K, Sanka R, Sheth N, Orvis J, Sodergren E, Wang Z, Weinstock GM, Mitreva M (2012). Optimizing read mapping to reference genomes to determine composition and species prevalence in microbial communities. PLoS ONE.

[R41] Maurice CF, Haiser HJ, Turnbaugh PJ (2013). Xenobiotics shape the physiology and gene expression of the active human gut microbiome. Cell.

[R42] Maynard CL, Elson CO, Hatton RD, Weaver CT (2012). Reciprocal interactions of the intestinal microbiota and immune system. Nature.

[R43] McHardy IH, Goudarzi M, Tong M, Ruegger PM, Schwager E, Weger JR, Graeber TG, Sonnenburg JL, Horvath S, Huttenhower C (2013). Integrative analysis of the microbiome and metabolome of the human intestinal mucosal surface reveals exquisite inter-relationships. Microbiome.

[R44] Meyer F, Trimble WL, Chang EB, Handley KM (2012). Functional predictions from inference and observation in sequence-based inflammatory bowel disease research. Genome Biol.

[R45] Molodecky NA, Panaccione R, Ghosh S, Barkema HW, Kaplan GG, Alberta Inflammatory Bowel Disease Consortium (2011). Challenges associated with identifying the environmental determinants of the inflammatory bowel diseases. Inflamm Bowel Dis.

[R46] Morgan XC, Tickle TL, Sokol H, Gevers D, Devaney KL, Ward DV, Reyes JA, Shah SA, LeLeiko N, Snapper SB (2012). Dysfunction of the intestinal microbiome in inflammatory bowel disease and treatment. Genome Biol.

[R47] Ordás I, Eckmann L, Talamini M, Baumgart DC, Sandborn WJ (2012). Ulcerative colitis. Lancet.

[R48] Ott SJ, Musfeldt M, Wenderoth DF, Hampe J, Brant O, Fölsch UR, Timmis KN, Schreiber S (2004). Reduction in diversity of the colonic mucosa associated bacterial microflora in patients with active inflammatory bowel disease. Gut.

[R49] Packey CD, Sartor RB (2009). Commensal bacteria, traditional and opportunistic pathogens, dysbiosis and bacterial killing in inflammatory bowel diseases. Curr Opin Infect Dis.

[R50] Presley LL, Ye J, Li X, Leblanc J, Zhang Z, Ruegger PM, Allard J, Mc-Govern D, Ippoliti A, Roth B (2012). Host-microbe relationships in inflammatory bowel disease detected by bacterial and metaproteomic analysis of the mucosal-luminal interface. Inflamm Bowel Dis.

[R51] Prince AL, Antony KM, Ma J, Aagaard KM (2014). The microbiome and development: a mother’s perspective. Semin Reprod Med.

[R52] Qin J, Li R, Raes J, Arumugam M, Burgdorf KS, Manichanh C, Nielsen T, Pons N, Levenez F, Yamada T (2010). A human gut microbial gene catalogue established by metagenomic sequencing. Nature.

[R53] Qin J, Li Y, Cai Z, Li S, Zhu J, Zhang F, Liang S, Zhang W, Guan Y, Shen D (2012). A metagenome-wide association study of gut microbiota in type 2 diabetes. Nature.

[R54] Ratner RE, Diabetes Prevention Program Research (2006). An update on the Diabetes Prevention Program. Endocr Pract.

[R55] Renz H, von Mutius E, Brandtzaeg P, Cookson WO, Autenrieth IB, Haller D (2011). Gene-environment interactions in chronic inflammatory disease. Nat Immunol.

[R56] Romero R, Hassan SS, Gajer P, Tarca AL, Fadrosh DW, Bieda J, Chaemsaithong P, Miranda J, Chaiworapongsa T, Ravel J (2014a). The vaginal microbiota of pregnant women who subsequently have spontaneous preterm labor and delivery and those with a normal delivery at term. Microbiome.

[R57] Romero R, Hassan SS, Gajer P, Tarca AL, Fadrosh DW, Nikita L, Galuppi M, Lamont RF, Chaemsaithong P, Miranda J (2014b). The composition and stability of the vaginal microbiota of normal pregnant women is different from that of non-pregnant women. Microbiome.

[R58] Savage DC (1977). Microbial ecology of the gastrointestinal tract. Annu Rev Microbiol.

[R59] Scanlan PD, Shanahan F, O’Mahony C, Marchesi JR (2006). Culture-independent analyses of temporal variation of the dominant fecal microbiota and targeted bacterial subgroups in Crohn’s disease. J Clin Microbiol.

[R60] Schloissnig S, Arumugam M, Sunagawa S, Mitreva M, Tap J, Zhu A, Waller A, Mende DR, Kultima JR, Martin J (2013). Genomic variation landscape of the human gut microbiome. Nature.

[R61] Shu CJ, Benoist C, Mathis D (2012). The immune system’s involvement in obesity-driven type 2 diabetes. Semin Immunol.

[R62] Sokol H, Lay C, Seksik P, Tannock GW (2008). Analysis of bacterial bowel communities of IBD patients: what has it revealed?. Inflamm Bowel Dis.

[R63] Sokol H, Seksik P, Furet JP, Firmesse O, Nion-Larmurier I, Beaugerie L, Cosnes J, Corthier G, Marteau P, Doré J (2009). Low counts of Faecalibacterium prausnitzii in colitis microbiota. Inflamm Bowel Dis.

[R64] Stappenbeck TS, Wong MH, Saam JR, Mysorekar IU, Gordon JI (1998). Notes from some crypt watchers: regulation of renewal in the mouse intestinal epithelium. Curr Opin Cell Biol.

[R65] Tong M, Li X, Wegener Parfrey L, Roth B, Ippoliti A, Wei B, Borneman J, McGovern DP, Frank DN, Li E (2013). A modular organization of the human intestinal mucosal microbiota and its association with inflammatory bowel disease. PLoS ONE.

[R66] Turnbaugh PJ, Ley RE, Mahowald MA, Magrini V, Mardis ER, Gordon JI (2006). An obesity-associated gut microbiome with increased capacity for energy harvest. Nature.

[R67] Turnbaugh PJ, Ridaura VK, Faith JJ, Rey FE, Knight R, Gordon JI (2009). The effect of diet on the human gut microbiome: a metagenomic analysis in humanized gnotobiotic mice. Sci Transl Med.

[R68] Walters TD, Kim MO, Denson LA, Griffiths AM, Dubinsky M, Markowitz J, Baldassano R, Crandall W, Rosh J, Pfefferkorn M (2014). Increased effectiveness of early therapy with anti-tumor necrosis factor-a vs an immunomodulator in children with Crohn’s disease. Gastroenterology.

[R69] Wang Q, Garrity GM, Tiedje JM, Cole JR (2007). Naive Bayesian classifier for rapid assignment of rRNA sequences into the new bacterial taxonomy. Appl Environ Microbiol.

[R70] Wijesinghe DS, Allegood JC, Gentile LB, Fox TE, Kester M, Chalfant CE (2010). Use of high performance liquid chromatography-electrospray ionization-tandem mass spectrometry for the analysis of ceramide-1-phosphate levels. J Lipid Res.

[R71] Wijesinghe DS, Mayton EK, Mietla JA, Mukherjee A, Wu J, Fang X, Chalfant CE (2011). Characterization of lysophosphatidic acid subspecies produced by autotaxin using a modified HPLC ESI-MS/MS method. Anal Methods.

[R72] Willing B, Halfvarson J, Dicksved J, Rosenquist M, Järnerot G, Eng-strand L, Tysk C, Jansson JK (2009). Twin studies reveal specific imbalances in the mucosa-associated microbiota of patients with ileal Crohn’s disease. Inflamm Bowel Dis.

[R73] Willing BP, Dicksved J, Halfvarson J, Andersson AF, Lucio M, Zheng Z, Järnerot G, Tysk C, Jansson JK, Engstrand L (2010). A pyrosequencing study in twins shows that gastrointestinal microbial profiles vary with inflammatory bowel disease phenotypes. Gastroenterology.

[R74] Wylie KM, Mihindukulasuriya KA, Sodergren E, Weinstock GM, Storch GA (2012a). Sequence analysis of the human virome in febrile and afebrile children. PLoS ONE.

[R75] Wylie KM, Weinstock GM, Storch GA (2012b). Emerging view of the human virome. Transl Res.

[R76] Xavier RJ, Podolsky DK (2007). Unravelling the pathogenesis of inflammatory bowel disease. Nature.

